# Interaction of hookworm 14-3-3 with the forkhead transcription factor DAF-16 requires intact Akt phosphorylation sites

**DOI:** 10.1186/1756-3305-2-21

**Published:** 2009-04-24

**Authors:** Joshua E Kiss, Xin Gao, Joseph M Krepp, John M Hawdon

**Affiliations:** 1 Department of Microbiology, Immunology, and Tropical Medicine and Department of Biological Sciences, The George Washington University, Washington, DC 20037, USA

## Abstract

**Background:**

Third-stage infective larvae (L3) of hookworms are in an obligatory state of developmental arrest that ends upon entering the definitive host, where they receive a signal that re-activates development. Recovery from the developmentally arrested dauer stage of *Caenorhabditis elegans *is analogous to the resumption of development during hookworm infection. Insulin-like signaling (ILS) mediates recovery from arrest in *C. elegans *and activation of hookworm dauer L3. In *C. elegans*, phosphorylation of the forkhead transcription factor DAF-16 in response to ILS creates binding cites for the 14-3-3 protein *Ce*-FTT-2, which translocates DAF-16 out of the nucleus, resulting in resumption of reproductive development.

**Results:**

To determine if hookworm 14-3-3 proteins play a similar role in L3 activation, hookworm FTT-2 was identified and tested for its ability to interact with *A. caninum *DAF-16 *in vitro*. The *Ac*-FTT-2 amino acid sequence was 91% identical to the *Ce*-FTT-2, and was most closely related to FTT-2 from other nematodes. *Ac*-FTT-2 was expressed in HEK 293T cells, and was recognized by an antibody against human 14-3-3β isoform. Reciprocal co-immunoprecipitations using anti-epitope tag antibodies indicated that *Ac*-FTT-2 interacts with *Ac*-DAF-16 when co-expressed in serum-stimulated HEK 293T cells. This interaction requires intact Akt consensus phosphorylation sites at serine107 and threonine312, but not serine381. *Ac*-FTT-2 was undetectable by Western blot in excretory/secretory products from serum-stimulated (activated) L3 or adult *A. caninum*.

**Conclusion:**

The results indicate that *Ac*-FTT-2 interacts with DAF-16 in a phosphorylation-site dependent manner, and suggests that *Ac*-FTT-2 mediates activation of L3 by binding *Ac*-DAF-16 during hookworm infection.

## Background

Hookworm infection is one of the most common chronic illnesses in the world, with an estimated 740 million infections [1]. The developmentally arrested third-stage infective larvae (L3) of hookworms resume development in response to a host-specific signal encountered during invasion. This cue initiates a signaling pathway that results in expression of genes required for development and molting to the L4 and subsequent adult stage. This signaling pathway represents a potential target for intervention in the hookworm life cycle as a means of preventing infection.

When non-feeding arrested L3 of the canine hookworm *Ancylostoma caninum *are incubated with serum components *in vitro*, they resume feeding within 12 hrs [2,3]. While they do not molt *in vitro*, "activation" by serum induces the release of molecules associated with infection, including proteases and activation-associated secretory proteins (ASPs) [4-7], and differential gene expression [8]. Hookworm L3 activation is mediated by an insulin-like signaling (ILS) pathway, similar to recovery from the analogous dauer stage of the free-living nematode *Caenorhabditis elegans *[9,10]. This dauer stage is morphologically, behaviorally, and functionally analogous to the hookworm L3, and recovery from it has been proposed as a model for the hookworm infective process [11,12]. The molecular biology of dauer recovery is well-defined, and has provided a useful framework for investigation of the molecular biology of hookworm infection.

Recovery from dauer in *C. elegans *is mediated by an insulin-like signaling (ILS) pathway [13]. Replete environmental conditions initiate a signaling cascade through an insulin growth factor 1 (IGF-1)-like receptor (encoded by the *daf-2 *gene), a phosphatidylinositol 3-kinase (PI3-K, *age-1*), and protein kinase B/Akt [14-20]. One of the key downstream targets of ILS is the forkhead/FOXO transcription factor DAF-16. Under dauer-inducing conditions, DAF-16 is located primarily in the nucleus, where it binds to target genes and mediates entry into and maintenance of dauer [15,21]. ILS during recovery causes the phosphorylation of DAF-16 by the serine/threonine kinase Akt [18], thereby creating binding sites for 14-3-3 proteins [22]. Binding of 14-3-3 to phosphorylated DAF-16 results in translocation of DAF-16 from the nucleus to the cytoplasm, and expression of genes associated with growth and development [23,24].

The 14-3-3 proteins are highly conserved ~30 kDa acidic dimeric proteins found in all eukaryotes. They function in cell signaling, cell cycle regulation, intracellular trafficking, and other process by modulating protein-protein interactions [22]. Typically organisms contain several isoforms, and homo- or heterodimers bind to phosphoserine- or phosphothreonine-containing motifs [25]. All forms of 14-3-3 share a similar tertiary structure, the dimer contact residues are highly conserved, and each monomer is capable of binding a phosphopeptide independently [25]. There are two isoforms of 14-3-3 in *C. elegans*, *ftt-2 *and *par-5*, but only *ftt-2 *is involved in regulation of DAF-16. Specific knockdown by RNAi of *ftt-2*, but not *par-5*, caused increased transcription of DAF-16 target genes [26].

Activation of hookworm L3 requires ILS [13,27], and recently the ortholog of *Ce*-DAF-16 was identified from the hookworms *A. caninum *and *A. ceylanicum *and shown to bind to a conserved DNA binding element [28]. Given the conservation of this pathway and the similarity between L3 activation and dauer recovery, we asked whether *Ac*-DAF-16 was regulated by a 14-3-3 protein as well. We report here that the ortholog of *Ce*-FTT-2 from *A. caninum *interacts with *Ac*-DAF-16 *in vivo*, and that this interaction requires intact Akt phosphorylation sites on DAF-16. This provides further support for the use of dauer recovery as a model for larval activation during the hookworm infective process.

## Results

### Cloning and characterization of Ac-ftt-2

A consensus sequence of a cluster of eight *A. caninum *expressed sequence tag (EST) sequences homologous to 14-3-3 proteins was used to design oligonucleotide primers to isolate the complete *Ac-ftt-2 *cDNA from *A. caninum*. Using a hemi-nested strategy, the 5' end was isolated using the conserved nematode spliced leader sequence [29] as the forward primer, coupled with two nested gene-specific reverse primers in successive PCR reactions. The second reaction produced an amplicon of approximately 600 bp that contained the SL sequence and overlapped the 5' end of the *A. caninum *EST sequence. A similar strategy was used to isolate the 3' end from an *A. caninum *cDNA library. A reverse primer complementary to the flanking T7 sequence in the vector was used with two nested forward primers to generate an amplicon of approximately 700 bp that contained a poly d(A) tail, and overlapped the EST and the 5' end sequences. New primers were designed to amplify the entire coding sequence of *A. caninum *14-3-3 cDNA, which was cloned into pET28 vector and confirmed by DNA sequencing of both strands. The full-length cDNA sequence was deposited in GenBank (accession number FJ842376).

The full-length 14-3-3 cDNA contained the conserved 22 nucleotide nematode SL at the 5' end [30], which was followed by 33 untranslated nucleotides and an ATG codon encoding the starting methionine at nucleotide 56. The cDNA is predicted to encode an open reading frame of 249 amino acids, ending with a TAA termination codon at nucleotide 803, and followed by a 327 bp 3'-untranslated region. A canonical AATAAT polyadenylation signal [31] was located 11 bp upstream of the poly (dA) tail (nucleotides 1116 to 1121). The deduced amino acid sequence has a predicted mass of 28174 Da and a calculated pI of 4.87. The hookworm 14-3-3 lacks a secretory signal peptide [32], and contains a nuclear export signal (LxxxLxL) [33] at amino acids 223 to 229, consistent with a role in nuclear transport [34].

**Figure 1:**
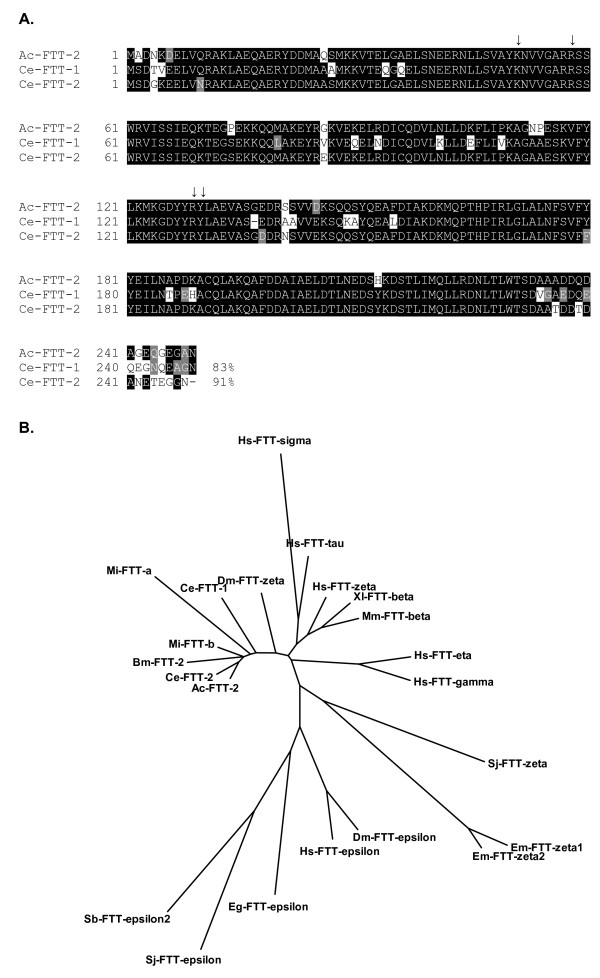
**The phylogenetic relationship between *Ac*-FTT-2 and selected 14-3-3 protein family members**. **A**. Alignment of *Ac*-FTT-2 with *C. elegans *14-3-3 proteins. Shading indicates residues that are identical (black) or similar (gray) to *Ac*-FTT-2. Amino acid residues that directly contact phosphate groups of targets are marked with an arrow. Protein sequences were aligned using CLUSTAL W software and displayed using BOXSHADE software located on the Swiss EMBnet server [74]. **B**. Neighbor joining tree of representative 14-3-3 proteins. Proteins were aligned using CLUSTAL W software on the Swiss EMBnet server [74]. Amino acid distances were calculated using the Poisson correction model in the MEGA program version 3.1 [75]. Major bootstrap values (1000 replications) are shown at each node. *Ac, A. caninum; Bm. Brugia malayi; Ce, Caenorhabditis elegans; Dm, Drosophila melanogaster; Eg, Echinococcus granulosus; Em, Echinococcus multilocularis; Hs, Homo sapiens*; *Mi, Meloidogyne incognita; Sb, Schistosoma bovis; Sj, Schistosoma japonicum, Xl, Xenopus laevis*. Accession numbers: *Ce*-FTT-1, [Genbank:CAA98138]; *Ce*-FTT-2, [Genbank:CAA91474]; *Bm*-FTT-2, [Genbank:XP_001895095]; *Dm*-FTT-ε, [Genbank:P92177]; *Dm*-FTT-ζ, [Genbank:P29310]; *Eg*-FTT-ε, [Genbank:AAX73175]; *Em*-FTT-ζ1, [Genbank:AAC48315]; *Em*-FTT-ζ2, [Genbank:AAM94864]; *Hs*-FTT-γ, [Genbank:P61981]; *Hs*-FTT-ε, [Genbank:P62258];*Hs*-FTT-ζ, [Genbank:P63104];*Hs*-FTT-η, [Genbank:Q04917];*Hs*-FTT-σ, [Genbank:P31947];*Hs*-FTT-τ, [Genbank:P27348]; *Mi*-FTT-a, [Genbank:AAL40719]; *Mi*-FTT-b, [Genbank:AAR85527]; *Mm*-FTT-β, [Genbank:Q9CQV8]; *Sb*-FTT-ε2, [Genbank:AAT39381]; *Sj*-FTT-ζ, [Genbank:AAD56715]; *Sj*-FTT-ε, [Genbank:AAC62003]; *Xl*-FTT-β, [Genbank:Q5XGC8].

A BLASTP search [35] of the non-redundant GenBank database using the deduced amino acid sequence confirmed that the hookworm protein was a member of the 14-3-3 protein family (Pfam PF00244, Interpro IPR000308). The best matches were to nematode 14-3-3 proteins, including *Caenorhabditis briggsae *(score 464, 2e-129), *C. elegans *(score 464, 4e-129). *C. brenneri *(score 462, 8e-129), and *Meloidogyne incognita *(score 461, 3e-128). While mammals have at least seven closely related isoforms [22], *C. elegans *has only two [26]. Alignment with *Ce*-FTT-1 (also known as PAR-5) and *Ce*-FTT-2 amino acid sequences shows that the hookworm 14-3-3 is more closely related to *Ce*-FTT-2 (91% identity) than *Ce*-FTT-1 (83% identity) (Fig. 1A). Furthermore, phylogenetic analysis indicates the hookworm 14-3-3 clusters most closely with FTT-2 isoforms from several other nematodes, including *M. incognita *and *Brugia malayi *(Fig. 1B). Therefore, the hookworm 14-3-3 protein most likely represents the *Ce*-FTT-2 ortholog, and will be referred to as *Ac*-FTT-2.

The 14-3-3 proteins are a family of dimeric proteins that modulate protein-protein interactions [22]. Phosphorylation of the interacting protein at sequence-specific sites mediates its interaction with the 14-3-3. Each subunit of the 14-3-3 dimer can bind to a phosphoserine or phosphothreonine ligand independently [34]. The most highly conserved region, the peptide-binding pocket, contains the residues that contact the phosphorylated amino acids. These residues (Lys51, Arg58, Arg129 and Tyr130) are completely conserved in *Ac*-FTT-2 (Fig. 1A). The N-terminal portion of 14-3-3 proteins is required for dimerization, and contains two conserved phosphorylation sites (Ser59 and Ser65) that are substrates for several kinases in mammals, including Akt and protein kinase C (PKC) [22]. Phosphorylation of Ser59 converted 14-3-3 dimers to monomers [36], suggesting a possible regulatory mechanism. However, a c-Jun N-terminal kinase (JNK) site at amino acid 186 [22] is absent from both *Ac*-FTT-2 and *Ce*-FTT-2, but is conserved in PAR-5. This further supports the conclusion that *Ac*-FTT-2 represents the ortholog of *Ce*-FTT-2.

### Transfection and immunoprecipitation of Ac-FTT-2 from HEK 293 cells

**Figure 2:**
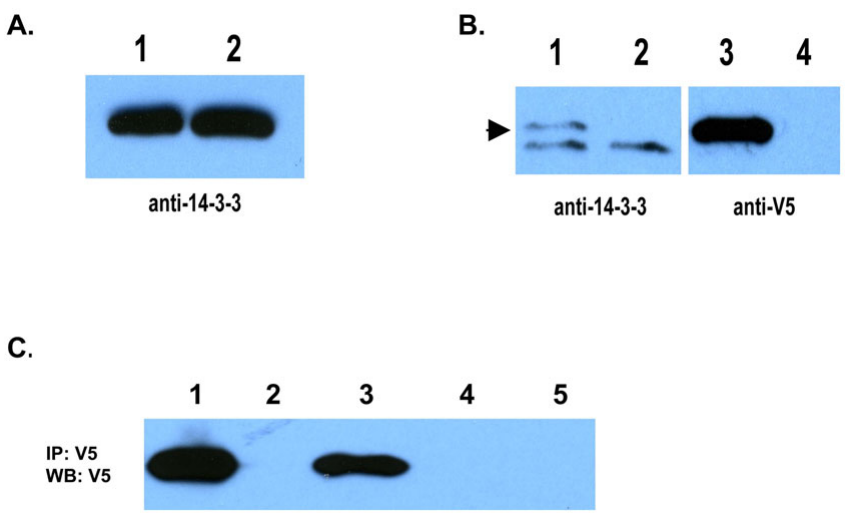
**Expression of *Ac*-FTT-2 and detection with anti-human 14-3-3β antibody**. **A**. Western blot of *A. caninum *L3 worm lysates. Soluble protein (5 μg) from 65,000 activated and non-activated L3 lysate was separated by 4–20% gradient SDS-PAGE, followed by Western blotting with anti-human 14-3-3β antibody. Lane 1, non-activated L3 extract; lane 2, activated L3 extract. **B**. Expression of *A. caninum *V5-tagged recombinant FTT-2 in human embryonic kidney 293 cells. A cDNA encoding full length *Ac-ftt-2 *was cloned into vector pcDNA3.1/V5-His and transfected into HEK293 cells for expression. After 48 h, 16 μg of total protein was separated by SDS-PAGE, and proteins detected by Western blotting with anti-human 14-3-3β antibody (left panel) or anti-V5 antibody (right panel). Lanes 1 and 3, *Ac*-FTT-2 transfected; lanes 2 and 4, mock transfected (empty vector).**C**. Immunoprecipitation of *A. caninum *V5-tagged recombinant FTT-2 expressed in human embryonic kidney 293 cells. Lysates were prepared 48 h after transfection with pcDNA3.1/V5-His/*Ac-ftt-2*. Mock lysates were made from cells transfected with the empty vector alone. Recombinant *Ac*-FTT-2 was precipitated by the addition of anti-V5 antibody. Bound sample refers to precipitated beads, and unbound refers to supernatant removed from precipitated beads. Lane 1, *Ac-ftt-2 *whole lysate only (input); lane 2, *Ac-ftt-2 *lysate unbound sample; Lane 3, *Ac-ftt-2 *lysate bound sample; Lane 4, mock lysate unbound sample; Lane 5, mock lysate bound sample.

Given the high level of conservation in 14-3-3 molecules, we tested whether an antibody against the human β isoform of 14-3-3 could detect *Ac*-FTT-2. As shown in Figure 2A, the 14-3-3β antibody recognized a single band in lysates of both untreated and serum-stimulated (activated) *A. caninum *L3 by Western blot. Next, we expressed recombinant *Ac*-FTT containing a V5 epitope tag in HEK293 cells. Western blotting of cell lysates indicated that *Ac*-FTT-2 was expressed in mammalian cells (Fig. 2B). An anti-V5 body recognized a single band of the appropriate size in lysates of cells transfected with pcDNA3.1V5/*Ac-ftt-2 *plasmid, but not in lysates from cells transfected with empty vector. The 14-3-3β antibody recognized two bands in the transfected cells, and a single band in the mock transfected cells. The lower molecular weight band, present in both transfected and mock cells, is endogenous 14-3-3, while the higher molecular weight band is the expressed FTT-2/V5 fusion protein, which migrates more slowly in the gel because of the attached epitope tag. This indicates that recombinant *Ac*-FTT-2 can be expressed in mammalian cells, and recognized by an antibody against the epitope tag and an anti-human 14-3-3 antibody.

Dauer recovery in *C. elegans *is mediated by 14-3-3 through regulation of DAF-16 subcellular localization and transcriptional activities [26]. This regulation is dependent on insulin-like signalling [23]. Because the resumption of development associated with infection in hookworms is analogous to dauer recovery [11,37], we hypothesized that DAF-16 is regulated by 14-3-3 in response to ILS as in dauer recovery in *C. elegans*. Previously, we demonstrated that *Ac*-DAF-16 binds to and drives transcription from a conserved hookworm DAF-16 DNA binding element in mammalian cells [38]. As hookworms have yet to be transfected with foreign DNA, we again used mammalian cells to examine interactions between *Ac*-FTT-2 and *Ac*-DAF-16.

We first tested whether we could immunoprecipitate recombinant V5-tagged *Ac*-FTT-2 from HEK293 cell lysates. As shown in Figure 2C, anti-V5 antibody successfully pulled down recombinant *A. caninum *FTT-2, as detected by Western blot with HRP-conjugated anti-V5 antibody (lane 3) and with anti-14-3-3β (not shown). *Ac*-FTT was not detected in unbound supernatant from the immunoprecipitate (Fig. 2C, lane 2), nor in control (empty vector) transfected cell lysate supernatant or immunoprecipitate (lanes 4 and 5, respectively), indicating that the ant-V5 antibody specifically precipitated the recombinant *Ac*-FTT-2.

After confirming that we could express and immunoprecipitate recombinant *Ac*-FTT, we next tested if *Ac*-FTT-2 and *Ac*-DAF-16 interact *in vivo*. In *C. elegans*, FTT-2 interacts with and regulates *Ce*-DAF-16 by excluding it from the nucleus [26]. HEK 293 cells were co-transfected with equal amounts of plasmid pcDNA3.1V5/*Ac-ftt-2*, encoding V5-tagged *Ac*-FTT-2, and plasmid pCMV4FLAG/*Ac-daf-16 *encoding FLAG-tagged *A. caninum *forkhead transcription factor DAF-16 [38]. After serum treatment for 24 hrs, reciprocal co-IP was performed on cell lysates using anti-V5 and anti-FLAG antibody resin.

**Figure 3:**
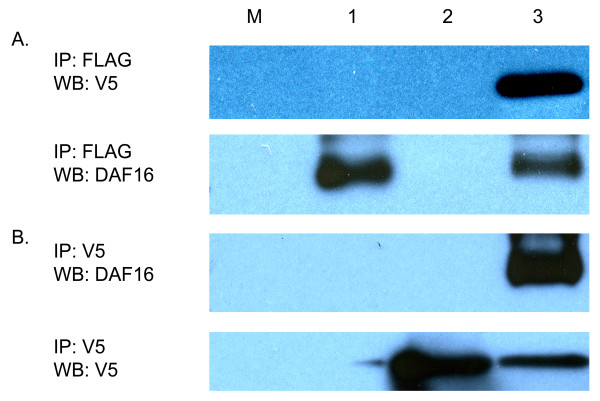
**Co-immunoprecipitation of FTT-2 and DAF-16 expressed in human embryonic kidney 293 cells**. Cells were transfected singly (4 μg) or co-transfected (2 μg each) with pcDNA3.1V5/*Ac-ftt-2 *and pCMV4FLAG/*Ac-daf-16*. At 16 hrs, the cells were incubated with 20% serum, and lysates prepared 24 hrs later. Lane M, cells transfected with empty pcDNA3.1/V5-His vector (mock); lane 1, cells transfected with *Ac-daf-16 *alone; lane 2, cells transfected with *Ac-ftt-2 *alone; lane 3, serum treated co-transfected cells. **A**. Immunoprecipitation with anti-FLAG (M2) agarose. *Top panel*, Western blot with anti-V5 antibody; *bottom panel*, the same blot stripped and probed with DAF-16 antiserum. **B**. Immunoprecipitation with anti-V5 agarose. *Top panel*, Western blot with DAF-16 antiserum; *bottom panel*, the same blot stripped and probed with V5 antibody.

As shown in Figure 3A, anti-FLAG agarose pulled down both a 64 kDa FLAG-tagged DAF-16 and the V5-tagged recombinant *Ac*-FTT-2 from co-transfected cells (lanes 3), but only recombinant DAF-16 from cells singly transfected with *Ac-daf-16 *(lane 1). As expected, anti-FLAG agarose did not pull down any protein from the *Ac-ftt-2 *singly transfected cells (lane 2). Conversely, anti-V5 agarose precipitated both proteins from co-transfected cells, but only recombinant 14-3-3 from cells singly transfected with the *Ac-ftt-2 *construct, and nothing from cells expressing recombinant *Ac*-DAF-16 only (Fig. 3B). This indicates that *Ac*-FTT-2 interacts with *Ac*-DAF-16 in mammalian cell culture, and suggests that a similar interaction occurs in worms.

### Intact phosphorylation sites are required for the FTT-2 – DAF-16 interaction

**Figure 4:**
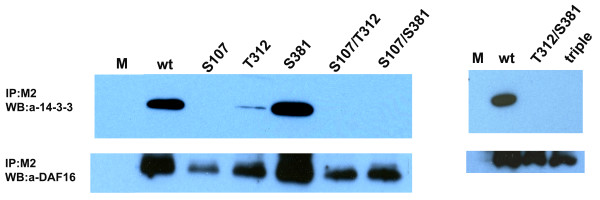
**Effect of phosphorylation site mutation on the interaction of *Ac*-FTT-2 with *Ac*-DAF-16**. HEK293 cells were co-transfected with 2 μg of pcDNA3.1V5/*Ac-ftt-2 *and 2 μg of wild-type or mutant pCMV4FLAG/*Ac-daf-16 *plasmids as above. Mock cells received 4 μg of empty pcDNA3.1/V5-His vector. Cell lysates were prepared 48 h after transfection, and then incubated with anti-FLAG (M2) agarose resin. Immunoprecipations were separated by 4–20% gradient SDS-PAGE and transferred to PVDF membrane. *Top panels*, Western blot with anti-human 14-3-3β antibody; *Bottom panels*, the blot was stripped and re-probed with *Ac*-DAF-16 antiserum. See text for description of the *Ac*-DAF-16 mutants.

Interaction of 14-3-3 with *Ce*-DAF-16 requires phosphorylation of specific serine or threonine residues by the protein kinase Akt (Cahill et al., 2001). To determine if the predicted Akt phosphorylation sites are important for the interaction of hookworm 14-3-3 and DAF-16, recombinant *Ac*-DAF-16 proteins with the phosphorylation sites mutated to alanine were tested for their ability to co-IP recombinant FTT-2 when co-expressed in HEK293 cells. Anti-FLAG M2 resin co-immunoprecipitated recombinant *Ac*-FTT-2 and wild type FLAG-tagged DAF-16 from cells expressing both proteins (Figure 4). However, mutation of serine107 to alanine (S107A) completely abolished the interaction with *Ac*-FTT-2, either singly or in combination with mutations at the other Akt phosphorylation sites (S107A:T312A, S107A:S381A, or triple). Mutation of threonine 312 (T312A) alone or in combination with mutations at the other sites also severely diminished the interaction with FTT-2 However, mutation of serine 381 (S381A) had no effect on the interaction with 14-3-3 (Figure 4). These data indicate that serine107 and threonine312 are essential for the interaction of *Ac*-DAF-16 with *Ac*-FTT-2.

### Secretion of Ac-FTT-2

**Figure 5:**
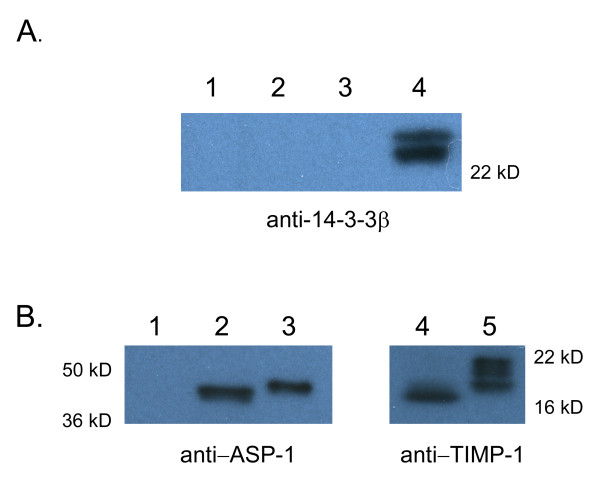
**Absence of *Ac*-FTT-2 in excretory/secretory products of adult and L3 *Ancylostoma caninum***. ESP from 6000 non-activated or activated L3, and 10 μg of adult ESP were separated by 4–20% gradient SDS-PAGE and transferred to PVDF membrane for Western blotting. **A**. Lane 1, non-activated L3 ESP; lane 2, activated L3 ESP; lane 3, adult ESP; lane 4, lysate (20 μg) of HEK293 expressing *Ac*-FTT-2. The blot was probed with anti-14-3-3β antibody. **B**. Control gels. Lane 1, non-activated L3 ESP; lane 2, activated L3 ESP; lane 3, recombinant ASP-1 (80 ng); lane 4, adult ESP; recombinant TIMP-1 (130 ng). Blots were probed with anti-ASP-1 antiserum (left) or anti-TMP-1 antiserum (right).

Hookworm L3 activated *in vitro *by incubation with serum components under host-like conditions release multiple proteins, including the activation associated secretory proteins 1 and 2 (ASP-1 and ASP-2) [5,6] and a metalloprotease [4]. Adult worms release molecules associated with feeding and survival in the host intestine, including anticoagulants [39], ASPs [40] and protease inhibitors [41]. To determine if *Ac*-FTT-2 was secreted, we took advantage of the cross-reactivity with the anti-human 14-3-3β antibody to examine ESP from L3 and adult stages by Western blot. As seen in Figure 5A, 14-3-3β antibody failed to detect any bands in non-activated, activated L3, or adult ESP (lanes 1–3), but recognized recombinant *Ac*-FTT-2 expressed in HEK293 cells (lane 4). As a control, L3 ESP were probed with ASP-1 antiserum, which detects a band in activated but not non-activated ESP (Figure 5B, lanes 1–3) [5]. Furthermore, antiserum against *Ac*-TMP-1, known to be released in adult ESP [42], detected *Ac*-TMP in the adult ESP (Figure 5B, lanes 4 and 5), indicating that the ESP contained secreted proteins. These data indicate that *Ac*-FTT-2 is not released in detectable amounts by either activated L3 or adult *A. caninum*.

## Discussion

When hookworm L3 infective stage larvae encounter and invade a permissive host, developmental pathways are initiated by host-specific signals and lead to the maturation of larvae into adult parasites. The molecular details of this process are unknown, but clues to hookworm developmental processes have been revealed by studies of the related free-living nematode *C. elegans*. Specifically, dauer recovery in *C. elegans *has been compared to the transition to parasitism in hookworms [11,37], and they share several conserved pathways and molecules [27,38,43,44]. Despite these similarities, detailed mechanistic knowledge of the molecular biology of the transition to parasitism is difficult to obtain due to the inability of hookworms to complete their life cycle *in vitro*. For this reason, the regulation and function of hookworm molecules must be studied indirectly, using heterologous systems. This and a previous study [38] represent the first applications of a mammalian cell culture system to investigate hookworm molecular biology. This system will allow us to investigate molecular mechanisms of hookworm infection for the first time.

Dauer recovery in *C. elegans *is mediated by ILS signaling in response to improving environmental conditions [13]. ILS activates the serine/threonine kinase Akt/PKB, which in turn phosphorylates the forkhead transcription factor DAF-16 on multiple serine/threonine residues [18]. Phosphorylation of DAF-16 creates binding sites for the 14-3-3 family protein FTT-2 and translocates DAF-16 to the cytoplasm [23,26], thereby allowing a pattern of gene expression associated with reproductive development. Previously, we isolated an ortholog of DAF-16 from the hookworm *A. caninum*, and demonstrated that it bound to and drove transcription from a conserved DAF-16 binding element when expressed in mammalian cells [38]. The similarity between *A. caninum *and *C. elegans *ILS and recovery from dauer arrest led us to hypothesize that ILS plays a similar role in hookworms. To investigate the regulation of *Ac*-DAF-16 by 14-3-3, we isolated the FTT-2 ortholog from *A. caninum*, and co-expressed it with *Ac*-DAF-16 in HEK293 cells

Most organisms contain multiple isoforms of 14-3-3, including seven in mammals, two in *Drosophila *and yeast, and 15 in plants [45]. Several isoforms have been identified in platyhelminths of the genera *Echinococcus *and *Schistosoma *[46,47]. To date, no nematodes have been shown to encode more than two FTT-2 isoforms. Sequence comparison and phylogenetic analysis indicated that the hookworm 14-3-3 was most closely related to FTT-2 molecules from *C. elegans *and other nematodes. Despite 86% sequence identity between 14-3-3 isoforms FTT-2 and PAR-5 in *C. elegans*, only FTT-2 affects dauer recovery. RNAi knockdown of *Ce-ftt-2 *enhanced dauer formation at permissive temperatures in dauer constitutive *daf-2 *mutants, increased nuclear localization of DAF-16, and promoted transcription of several DAF-16 target genes [26]. The high level of sequence conservation of *Ac*-FTT-2 with *Ce*-FTT-2 suggested that this isoform was most likely to interact with *Ac*-DAF-16 in hookworms. While FTT-2 affects dauer formation in *C. elegans*, PAR-5 also weakly interacts with *Ce*-DAF-16, suggesting a possible role in aspects of DAF-16 function other than those listed above [26]. Indeed, over-expression of *Ce*-FTT-2 and PAR-5 extend lifespan in a DAF-16-dependent manner, and both interact with SIR-2.1 and DAF-16 in *C. elegans *[48]. While our data suggest that *Ac*-FTT-2 shares similar functions with *Ce*-FTT-2, we can not rule out a function in infection for another isoform of 14-3-3 in hookworms.

Once we identified the appropriate 14-3-3 ortholog from *A. caninum*, we introduced *Ac-ftt-2 *cDNA into mammalian expression vectors and expressed epitope tagged fusion protein in HEK293 cells. The native and recombinant hookworm FTT-2 protein expressed in HEK293 cells was recognized by an antibody against the β isoform of human 14-3-3. The antibody also detected an endogenous 14-3-3 protein in 293 cells, as indicated by the lower molecular weight band that is also present in cells transfected with empty vector. This antibody will permit further investigation of *Ac*-FTT-2 function *in vitro *and with native worm protein.

*Ce*-DAF-16 has been demonstrated to interact with 14-3-3 previously [26]. To determine if *Ac*-FTT-2 interacted with *Ac*-DAF-16, we co-expressed both proteins in HEK293 cells and performed reciprocal co-IPs using antibodies against the epitope tags on the recombinant proteins. We were able to successfully IP both proteins using both antibodies from serum stimulated cells, indicating that FTT-2 interacts with DAF-16.

14-3-3 proteins bind to the specific amino acid sequence motifs RSXp**S**XP and RXXXp**S**XP, where p**S **represents phosphoserine and X represents any amino acid [25,26]. RXRXXS is the consensus Akt phosphorylation site [23] and corresponds closely with the 14-3-3 binding sequence. Mammalian Akt phosphorylates FOXO proteins, the mammalian orthologs of DAF-16, at predicted 14-3-3 binding sites [49]. Using mutant DAF-16 molecules with altered phosphorylation sites, we demonstrated that serine 107 and threonine 312 are essential for the FTT-2 interaction with DAF-16. Both serine 107 and threonine 312 are in predicted 14-3-3 binding motifs. Typically, FOXO family proteins have three Akt phosphorylation sites, with sites P1 and P2 flanking the DNA binding domain in the N-terminal half of the molecule, and site P3 in the C-terminal half. In FOXO-4, as in *Ac*-DAF-16, the P1 and P2 sites are required for interaction with the 14-3-3 protein [49]. Binding of the 14-3-3 to these sites is believed to mask the DNA binding domain, thereby interrupting the FOXO/DAF-16 function. Interestingly, this differs from the FTT-2/DAF-16 interaction in *C. elegans*, where the analogous N-terminal P1 site and the C-terminal P3 site are required for interaction [23]. Our results demonstrate the requirement of Akt phosphorylation sites for 14-3-3/DAF-16 association, and suggest that *Ac*-14-3-3 binds to phosphorylated *Ac*-DAF-16 to mediate ILS, as occurs in *C. elegans*. Further investigations are underway to confirm this hypothesis.

14-3-3 proteins have been reported from a growing number of helminth parasites. In nematodes, aside from *C. elegans *and now *A. caninum*, the only other well characterized 14-3-3 proteins are from the root-knot nematode *Meloidogyne incognita *[50]. Partial sequences from EST databases have been reported from *Strongyloides stercoralis*, *Haemonchus contortus *[46], and the hookworm of humans *Necator americanus *[51]. A 29 kDa protein in ESP from the adult stage of the ovine stomach worm *Teladorsagia circumcincta *was identified as a 14-3-3 protein [52]. The complete descriptive and functional characterization of these proteins remains to be performed.

More is known about the 14-3-3 proteins of platyhelminths. Multiple isoforms and sub-isoforms have been identified and characterized in *Schistosoma *and *Echinococcus *species [46,47]. In *S. mansoni*, 14-3-3ε-1 interacts with the TGF-β Type 1 receptor and enhances TGF-β signaling [53]. TGF-β signaling is required for dauer formation (although not recovery) in *C. elegans *[13,54], and the TGF-β receptor ligand DAF-7 has been identified from *A. caninum *[44]. Also, TGF-β signaling has been implicated in the reactivation of tissue-arrested *A. caninum *L3 [55]. Demonstration of a functional role for *Ac*-FTT-2 in TGF-β signaling awaits further characterization of this pathway in hookworms.

The 14-3-3 proteins of platyhelminths are associated with the tegument, or are actively secreted, making them available as targets of the host immune system and therefore potential vaccine antigens [47,56]. In *S. mansoni*, all three isoforms of 14-3-3 are expressed during infection and induce antibody production by the host [57]. The 14-3-3ζ isoform has shown some protection against schistosome challenge infections, eliciting worm reductions ranging from 25 to 65% [58-61]. The greatest success of a 14-3-3 as an anti-parasite vaccine antigen was in *Echinococcus multilocularis*, where vaccination with the 14-3-3ζ isoform resulted in a 97% reduction in parasite load following challenge in a mouse [47,62]. Recombinant 14-3-3ζ reduced nitric oxide production from activated macrophages *in vitro *[63], and may contribute to the immunosuppressive effect associated with alveolar cyst infection [47,64].

The FTT-2 ortholog in the root-knot nematode *M. incognita *is synthesized in the dorsal esophageal gland cell and present in stylet secretions [50,65]. In this parasite, stylet secretions play a role in the induction and maintenance of the feeding site within the plant host [66]. 14-3-3 proteins in ESP suggest an extra-corporeal function, including potentially influencing host immune responses [47,64]. Hookworms are known to release abundant ESP from both activated L3 [4-6] and adult stages [41,42]. Minimally, release of 14-3-3 by a life history stage would be a prerequisite for a vaccine antigen. However, we could not demonstrate *Ac*-FTT-2 in either larval or adult ESP, indicating that unlike some other helminth 14-3-3 proteins, *Ac*-FTT-2 is not secreted. Therefore it is unlikely that this protein interacts with the host immune system. It is more likely that it is part of endogenous signaling pathways, including ILS downstream of Akt.

## Conclusion

In conclusion, we report the identification and cloning of a 14-3-3 protein family member, *Ac*-FTT-2, from the canine hookworm *A. caninum*. *Ac*-FTT-2 is most closely related to *Ce*-FTT-2, a protein that mediates the effects of ILS in *C. elegans*. We demonstrated that *Ac*-FTT-2 interacts with the forkhead transcription factor Ac-DAF-16 when co-expressed in mammalian cells, and that this interaction requires intact Akt phosphorylation sites at serine 107 and threonine 312. Our data suggest that, as in *C. elegans *dauer recovery, the interaction of *Ac*-FTT-2 with phosphorylated *Ac*-DAF-16 mediates the effects of ILS on development, and therefore might play an important role in the hookworm infective process. Further investigations into the role of FTT-2 and DAF-16 in hookworm recovery will provide insights into the early, critical events of hookworm infection, and possibly novel intervention strategies for the prevention of hookworm disease.

## Methods

### Parasites

The Baltimore strain of *A. caninum *(U.S. National Parasite Collection accession 100655.00) was maintained in beagles as described previously [67]. The beagles were housed and treated according to a protocol approved by the George Washington University Institutional Care and Use Committee. Infective L3 were recovered from coproculture by a modified Baermann technique and stored at room temperature in buffer BU (50 mM Na_2_HPO_4_/22 mM KH_2_PO_4_/70 mM NaCL, pH 6.8) for up to one month [68], or snap-frozen in liquid N_2 _and stored at -80 C until lysates were prepared.

### In vitro larval activation and collection of excretory/secretory products

*Ancylostoma caninum *L3 were activated under host-like conditions as described previously [6]. Briefly, approximately 5000 decontaminated L3 were incubated at 37C, 5% CO_2 _for 24 hr in 0.5 ml RPMI_1640 _tissue culture medium supplemented with 25 mM HEPES (pH 7.0) and antibiotics (RPMI-c) in individual wells of 24-well tissue culture plates. L3 were activated by the addition of 10% (v/v) of a less than 10 kDa ultrafiltrate of canine serum and 15 mM S-methyl-glutathione (GSM, Sigma Chemical, St. Louis, MO) in RPMI-c. Non-activated L3 were incubated with RPMI-c medium alone. The percentage of L3 feeding (or "activated") was determined as described elsewhere [2]

Following incubation, medium containing L3 was transferred to microcentifuge tubes and centrifuged at 13,000 rpm for 5 min. The supernatant containing the excretory/secretory products (ESP) was collected and concentrated by ultrafiltration using Centricon YM-10 cartridge (Millipore). The retentate was washed with 0.5 mL PBS, and concentrated 10-fold for electrophoresis.

### Ac-FTT-2 amplification and cloning

A consensus sequence derived from a cluster of eight expressed sequence tags encoding an *A. caninum *14-3-3 (AC01065) [69] was used to design specific forward and reverse oligonucleotide primers for PCR. The *Ac*-*ftt-2 *cDNA ends were amplified in two separate PCR reactions. For the 5' end, the reverse primer R2 (5'-AAATTGAGAGCGAGGCCAAGGCG-3'), the forward nematode spliced leader primer SL (5'-GGTTTAATTACCCAAG TTTGAG-3') and first strand cDNA [70] were incubated in a PCR reaction for 35 cycles of 1 min at 94 C, 1 min at 57 C, and 2 min at 72, followed by a final extension for 4 min at 72 C.

The 3' end was isolated using a nested strategy from an *A. caninum *L3 directional cDNA library constructed in Lambda ZAP II [71]. The first reaction employed the forward primer FTT-F1 (5'-GAACTCGTGTTGAGGGCCAAGC-3') together with a primer complementary to the T7 promoter site in the vector flanking the 3' end of the cDNA insert site (5'-TAATACGACTCACTATAG-3'). The primers and 1 μl of cDNA library were incubated in a PCR reaction of 1 min at 94 C, 1 min at 55 C, and 2 min at 72 C, and a final extension for 5 min at 72 C. The first round reaction was diluted 1:10 and used as template in a second reaction containing the nested forward primer FTT-F2 (5'-CGAGCTGGAGAGTTATTT CGTCG-3') and reverse primer T7-NEST (5'-ACTCACTATAGGGCGAATTG-3'), using identical cycling conditions. Amplicons of approximately 550 bp (5' end) and 900 bp (3'end) were gel purified, cloned using the pGEM-T Easy TA cloning kit (Promega, Madison, WI), and the DNA sequence of both strands determined. The DNA sequences of the 5' and 3' end clones were aligned and combined to give the 1165-bp full-length composite sequence of *Ac-ftt*-2, including a 32-bp poly-d(A) tail.

The composite sequence was used to design primers to amplify and clone the full-length coding sequence. Forward primer FTT-FX (5'-TTAGGATCCATG GCCGAT AACAAGGATGAACTCG-3') containing a 5'*Bam*HI restriction site (underlined) and FTT-RX (5'-GATCTCGAGGAATCAGATCATA TGGGTTTAATTGGC-3') with a 5' *Xho*I restriction site were incubated in a PCR with *A. caninum *L3 first strand cDNA for 35 cycles of 95 C for 1 min, 55 C for 1 min, and 72 C for 2 min, followed by a final 5 min extension at 72 C. The amplicon was digested for 18 h at 37 C with 20 units each of *XhoI *and *BamHI *restriction enzymes (New England Biolabs, Beverley, MA) in *BamHI *buffer, gel purified and ligated into pET28a (Novagen, Gibbstown, NJ) which had been digested with the same enzymes. The ligation products were transformed into *E. coli *DH5α competent cells by standard methods, and the constructs confirmed by DNA sequencing.

The full length coding sequence of *A. caninum *14-3-3 cDNA (*Ac-ftt-2*) in pET28a was used as a template to amplify the insert for subcloning into a mammalian expression vector. Forward primer FTT-FX and reverse primer FTT-RX2 (5'-ATAACTCGAGAT TGGCAC CCTCTCCTTGC-3') containing a *XhoI *site were incubated in a PCR reaction with 12 ng of plasmid DNA for 35 cycles of 95 C for 1 min, 55 C for 1 min, and 72 C for 2 min, followed by a 5 min extension at 72 C. An amplicon of approximately 700 bp was purified by Nucleospin column (ClonTech, Palo Alto, CA), and 2.5 μg of the product digested with *XhoI *and *BamHI *restriction enzymes as above. The gel purified insert was ligated into the mammalian expression vector pcDNA3.1-V5/His (Invitrogen, Carlsbad, CA) cut with the same enzymes, and the ligation products were transformed as above. Positive colonies were confirmed by colony PCR using FX and RX2 primers, and the DNA sequence of both strands determined. All DNA concentrations were determined spectrophotometrically (Nanodrop, Thermo Fisher Scientific, Waltham, MA).

### Transfection of human embryonic kidney 293 cells

Growing human embryonic kidney 293 (HEK293) cells were re-suspended in fresh, pre-warmed RPMI_1640 _containing 10% FBS (Biowhittaker, Walkersville, MD), 2 mM L-glutamine (Mediatech, Herndon, VA), 100 IU/mL penicillin (Mediatech, Herndon, VA), and 100 μg/ml streptomycin (Mediatech, Herndon, VA), and evenly distributed into individual wells of a 6-well tissue culture plate. Medium was added to a final volume of 2 mL in each well and the plate was incubated overnight at 37 C, 5% CO_2_. The medium from each well was then replaced with fresh pre-warmed RPMI without disturbing attached cells and incubated for 2 h at 37 C. The cells were transfected with 4 μg of pcDNA3.1V5/*Ac-ftt-2 *plasmid DNA or empty pcDNA3.1V5 vector (mock) using Metafectene (Bionex, Munich) and incubated at 37°C for 48 h. Following incubation, media were removed and 2 mL of lysis buffer (20 mM Tris-HCl pH 7.5, 50 mM NaCl, 5 mM CHAPS, 1% Triton X-100, and 0.1% SDS) were added to each well to lyse the cells. Cell lysates were vortexed for 1 minute in microcentrifuge tubes, then frozen overnight at -80 C. Samples were thawed, vortexed again and centrifuged at room temperature for 5 min at 2300 × *g*. The supernatants were collected and stored at -20 C. Total protein concentrations were determined by bicinchoninic acid method (Micro BCA, Thermo Fisher) according to the manufacturer's protocol.

Co-transfections were performed as above with 2 μg each of pcDNA3.1V5/*Ac-ftt-2 *and pCMV4FLAG/*Ac-daf-16 *encoding the *A. caninum *forkhead transcription factor DAF-16 [38], or 2 μg each of the expression constructs and the corresponding empty vector. Cells were incubated overnight, followed by 20% serum treatment for 24 h, and lysates prepared.

### Western Blot of HEK293 cell extracts

Western blotting with the specific anti-V5 antibody was used to determine if recombinant *Ac*-FTT-2 (rFTT-2) was expressed in HEK293 cells. Samples (16 μg of total protein) of mock and pcDNA3.1-V5-*ftt-2 *transfected cell lysates were separated in a 4–20% gradient pre-cast Novex Tris-glycine SDS polyacrylamide gel (Invitrogen). Separated proteins were transferred to a polyvinylidene fluoride (PVDF) membrane for 110 min at 22 V by standard methods [41]. Following transfer, the membrane was blocked in 15 mL of 5% non-fat dry milk (NFDM) in PBS-T (0.05% Tween-20) overnight at 4 C. Membranes were incubated with mouse anti-human 14-3-3β/HRP antibody (Santa Cruz Bioechnology, Santa Cruz, CA) (1:20,000) or anti-V5/HRP mouse antibody (Invitrogen) (1:5,000) in 1% NFDM in PBS-T for 2 h at 22 C. After 3 × 10 min washes in PBS-T and 2 × 10 min washes in distilled water, the membrane was incubated in Enhanced Chemoluminescence (ECL) solution (Thermo Fisher), and chemoluminescent immunoreactive bands were detected by exposure to radiographic film.

### Immunoprecipitation of recombinant Ac-FTT from HEK 293 cell lysate

To determine if we could immunoprecipitate hookworm recombinant *Ac*-FTT-2, 5 μL of anti-V5 antibody (Invitrogen) were incubated for 16 h at 4 C with lysates of mock or *Ac-ftt-2 *transfected HEK 293 cells in a total volume of 500 μL of PBS-T (0.1% Tween-20). Protein A-agarose (Invitrogen) was pre-cleared with PBS-T and incubated with the antibody-antigen complexes for 1.5 h at 22 C with rotation. Following centrifugation for 30 s at 2300 × *g*, the supernatant was removed and stored frozen as the unbound sample, and the agarose complexes washed by rotation 3 × 5 min with 1 mL PBS-T. The agarose-antibody-antigen complexes were separated by SDS-PAGE and visualized by Western blot with HRP conjugated α-human 14-3-3β antibody or α-V5/HRP as described above.

### Reciprocal co-immunoprecipitation with α-V5 and α-FLAG using HEK293 cell lysates

HEK293 cells co-transfected with *Ac-daf-16 *and *Ac-ftt-2 *were lysed with 200 μL of cold buffer containing 50 mM NaCl, 50 mM Tris-HCl pH7.5, 0.1% NP-40, 5 mM EDTA, 1× Phosphatase Inhibitor Cocktail II (Calbiochem), and 1× Halt Protease Inhibitor cocktail (Pierce). Lysates were centrifuged at 4 C for 10 min at 10,000 × *g*, and the protein concentration of the supernatants determined by Micro BCA. Forty μg of anti-FLAG M2 resin (Sigma, St. Louis, MO) was prepared according to manufacturer's directions, added to ~1 mg of cell lysates, and mixed by rotation overnight at 4 C. Tubes were centrifuged for 30 s at 8200 × *g *and washed 3 times in TBS (50 mM Tris HCl pH7.4, 150 mM NaCl). Co-immunoprecipitation (co-IP) complexes were separated by 4–20% gradient SDS-PAGE and transferred to PVDF membrane for 7.5 min in an iBlot apparatus (Invitrogen). Following blocking, the membrane was probed with anti-V5/HRP (1:5000 dilution) at 22 C for 2 h. The membrane was washed 3 times in PBS-T and 2 times in distilled deionized water (ddH_2_O), dried, and incubated with ECL solution as above. Following visualization, the membrane was stripped with Restore buffer (Pierce) and blocked overnight in 5% NFDM in PBS-T at 4 C. The stripped membrane was re-probed with a specific *Ac*-DAF-16 polyclonal antiserum (1:20,000 dilution) [38], and visualized as above. The reciprocal co-IP was performed as above, using anti-V5 resin (Sigma) to pull-down protein complexes, and anti-DAF-16/anti-rabbit IgG-HRP and anti-V5/HRP to probe the membranes.

### Effect of phosphorylation on Ac-FTT-2 interaction with Ac-DAF-16

To determine the importance of phosphorylation at the predicted Akt phosphorylation sites for the interaction between 14-3-3 and *Ac*-DAF-16, the serine or threonine residue at each site was changed to alanine by site-directed mutagenesis. Using a Quickchange Site-Directed mutagenesis kit (Stratagene, La Jolla, CA), mutant *Ac*-DAF-16 plasmids were constructed in which each site was mutated separately (S107A, T312A, S381A), in pairs (S107A/T312A; S107A/S381A; T312A/S381A), or at all three phosphorylation sites (3A). The mutated plasmid constructs were confirmed by DNA sequencing.

HEK293 cells were co-transfected with 2 μg of pcDNA3.1V5/*Ac-ftt-2 *and 2 μg of wild-type or mutant pCMV4FLAG/*Ac-daf-16 *as above. Mock cells received 4 μg of empty pcDNA3.1/V5-His vector. After 24 hrs incubation, the cells were fed with 20% serum and grown overnight. Following cell lysis, co-IPs were performed using anti-FLAG agarose, separated by electrophoresis, and visualized by Western blot with anti-V5/HRP antibody as above. After stripping, the gel was re-probed with DAF-16 antiserum to visualize *Ac*-DAF-16.

### Preparation of lysates from frozen *A. caninum *L3 larvae

*Ancylostoma caninum *L3 were activated by incubating with canine serum fractions and S-methylglutathione as described above [6]. Approximately 100,000 frozen non-activated and activated L3 *A. caninum *larvae were ground to fine powder with a sterile mortar and pestle pre-chilled with liquid N_2_. The powder was added to 500 μL of cold PBS plus 1% EDTA, 1% Halt Protease Inhibitor Cocktail, and 1% Phosphatase Inhibitor Cocktail II and mixed by inversion. The lysates were centrifuged at 16,100 × *g *for 6 min at 4 C. Aliquots of the supernatants were removed to determine protein concentration by Micro BCA, and the remaining supernatants were stored at -20 C until needed.

### Secretion of *Ac*-FTT-2

To determine if *Ac*-FTT-2 was secreted during L3 activation, excretory/secretory products (ESP) from 6000 non-activated and activated L3 were collected as described previously [6]. Adult *A. caninum *ESP, recombinant tissue inhibitor of metalloproteases (*Ac*-TMP-1), and anti-TMP-1 antisera were gifts of Dr. Bin Zhan. Adult ESP were collected following overnight incubation of adult worms *in vitro*. Lysates (20 μg) of HEK293 cells expressing recombinant *Ac*-FTT-2, 80 ng of recombinant ASP-1 [5], and 130 ng of recombinant TMP-1 [42] were included as positive controls. Samples were separated at 140 V, 40 mA for 1.5 h in a 4–20% gradient pre-cast Novex Tris-glycine SDS polyacrylamide gel (Invitrogen), and transferred to PVDF membrane for 7.5 min in an iBlot apparatus (Invitrogen). Blots were incubated with ASP-1 antiserum (1:4000), TMP-1 antiserum (1:5000), or 14-3-3β antibody (1:20,000), respectively, and visualized using ECL Plus as described above.

### Sequence Analysis

Dideoxy DNA sequencing using the ABI BigDye Terminator Cycle Sequencing Ready Reaction Kit v3.1 was performed by the Nevada Genomics Center [72], and the reactions run on an ABI3730 DNA Analyzer. Sequence analysis was performed using BioEdit Sequence Alignment Editor version 5.0.9 [73], and multiple alignments performed using CLUSTAL W at Swiss EMBnet [74]. Neighbor-joining trees were constructed using MEGA version 3.1 [75]. Homology searches were done using BLASTP [35,76] at the National Center for Biotechnology Information [77] and WU-BLAST Parasite Genome Database Query at the European Bioinformatics Institute [78]. Percentage identity and similarity was determined using the BLASTP algorithm with the BLOSUM62 matrix. Conserved motifs were identified by searching Scansite at MIT [79] using MotifScan software [80], and nuclear export signals identified by searching the NetNES 1.1 server [33,81] at the Center for Biological Sequence Analysis, Technical University of Denmark.

## Competing interests

The authors declare that they have no competing interests.

## Authors' contributions

JEK participated in the study design, made the expression constructs, conducted the cell culture experiments, and drafted the manuscript. XG constructed the DAF-16 mutants, conducted the secretion experiments, and helped coordinate the study. JMK isolated and cloned *Ac*-FTT-2. JMH conceived of, designed, and coordinated the study, and polished the manuscript. All authors read and approved the final manuscript.
